# Neonatal Brains Exhibit Higher Neural Reparative Activities than Adult Brains in a Mouse Model of Ischemic Stroke

**DOI:** 10.3390/cells13060519

**Published:** 2024-03-15

**Authors:** Ryo Nishiyama, Takayuki Nakagomi, Akiko Nakano-Doi, Yoji Kuramoto, Masahiro Tsuji, Shinichi Yoshimura

**Affiliations:** 1 Institute for Advanced Medical Sciences, Hyogo Medical University, 1-1 Mukogawacho, Nishinomiya 663-8501, Japan; ds2142@hyo-med.ac.jp (R.N.); nakano@hyo-med.ac.jp (A.N.-D.); 2 Department of Neurosurgery, Hyogo Medical University, 1-1 Mukogawacho, Nishinomiya 663-8501, Japan; yo-kuramoto@hyo-med.ac.jp (Y.K.); hyogoneuro@yahoo.co.jp (S.Y.); 3 Department of Therapeutic Progress in Brain Diseases, Hyogo Medical University, 1-1 Mukogawacho, Nishinomiya 663-8501, Japan; 4 Department of Food and Nutrition, Kyoto Women’s University, 35 Kitahiyoshi-cho, Imakumano, Higashiyama-ku, Kyoto 605-8501, Japan; tsujima@kyoto-wu.ac.jp

**Keywords:** neonatal brain, adult brain, ischemic stroke, neural stem cell, neurogenesis, neural regeneration

## Abstract

The neonatal brain is substantially more resistant to various forms of injury than the mature brain. For instance, the prognosis following ischemic stroke is generally poor in the elderly but favorable in neonates. Identifying the cellular and molecular mechanisms underlying reparative activities in the neonatal brain after ischemic injury may provide feasible targets for therapeutic interventions in adults. To this end, we compared the reparative activities in postnatal day 13 and adult (8–12-week-old) mouse brain following middle cerebral artery occlusion. Immunohistochemistry revealed considerably greater generation of ischemia-induced neural stem/progenitor cells (iNSPCs) expressing nestin or Sox2 in ischemic areas of the neonatal brain. The iNSPCs isolated from the neonatal brain also demonstrated greater proliferative activity than those isolated from adult mice. In addition, genes associated with neuronal differentiation were enriched in iNSPCs isolated from the neonatal brain according to microarray and gene ontogeny analyses. Immunohistochemistry further revealed considerably greater production of newborn doublecortin^+^ neurons at the sites of ischemic injury in the neonatal brain compared to the adult brain. These findings suggest that greater iNSPC generation and neurogenic differentiation capacities contribute to the superior regeneration of the neonatal brain following ischemia. Together, our findings may help identify therapeutic targets for enhancing the reparative potential of the adult brain following stroke.

## 1. Introduction

Ischemic stroke is the primary cause of severe acute brain damage among the elderly, and long-term survival is generally poor [1,2,3]. Moreover, elderly stroke survivors are often left with irreversible sequelae associated with local brain damage. Stroke also occurs in neonates (within 28 days after birth), resulting in neurological dysfunction and neuropathology, as revealed by neuroimaging [4]. Although the incidence of stroke in newborns was reported to be only approximately 1/4000 live births [5], a recent study pointed out that it may actually be higher [6]. Neonatal stroke conditions include brain hemorrhage and cerebral venous sinus thrombosis [7], but the largest proportion (approximately 80%) are attributed to ischemic artery stroke, which mainly affects the middle cerebral artery (MCA) [7]. In contrast to stroke in the elderly, mortality following neonatal stroke is very low, and most patients are left with few if any complications [8,9]. In fact, the prognosis is even more favorable than following stroke during childhood or adulthood [10]. Therefore, identifying the neural mechanisms underlying enhanced stroke damage resistance in the neonatal brain may provide clues to more effective therapeutic targets for adult patients with stroke.

Although the repair capacity of the mature brain is limited, neural stem/progenitor cells (NSPCs) with multilineage differentiation potential are present in some brain regions throughout life [11]. Previous studies of mice expressing green fluorescent protein (GFP) under the control of the promoter for the NSPC marker nestin have shown that these cells are widely distributed during early brain development [12] but are restricted to specific neurogenic zones such as the subventricular zone (SVZ) and subgranular zone (SGZ) in the adult brain [12]. Kuhn and colleagues also reported that the proliferative potential of NSPCs decreases with age in rat brain [13]. These results suggest that greater numbers of NSPCs, more rapid proliferation, and (or) greater neuronal differentiation capacity may confer enhanced reparative potential in the neonatal brain compared to the mature brain.

We previously reported that regionally derived NSPCs are induced within and around injured regions after ischemic stroke in adult mice and that these injury/ischemia-induced NSPCs (iNSPCs) can contribute to tissue repair and neural regeneration in vitro and in vivo [14,15]. To examine if these cells contribute to the difference in stroke damage susceptibility between neonates and adults, we compared iNSPC activities and gene expression profiles between postnatal day 13 (P13) and adult mice (8–12 weeks old) following MCA occlusion (MCAO).

## 2. Materials and Methods

### 2.1. Induction of Ischemic Stroke in Mice

All animal housing and experimental procedures were approved by the Animal Care Committee of Hyogo Medical University (approval no. 21-006AG). Postnatal day 13 (P13) and 8-to-12-week-old wild-type mice (CB-17/Icr-+/+Jcl) were purchased from CLEA Japan (Tokyo, Japan). Permanent ischemic stroke was induced in neonatal [16] and adult mice [14,15,17], as previously described. Briefly, under isoflurane anesthesia, left MCAO was induced by electrocoagulation in adult and neonatal mice.

### 2.2. Ischemic Volume Evaluation

Ischemic volume was measured in the poststroke neonatal and adult mouse brains, as previously described [15]. Briefly, 1 day after MCAO, mice were anesthetized with a mixture containing medetomidine, midazolam, and butorphanol [14,17]. After removing the brains, 2 mm thick coronal sections were prepared. Sections were stained with 1% 2,3,5-triphenylteterazolium (TTC; Sigma-Aldrich, St. Louis, MO, USA) and subsequently fixed with 4% paraformaldehyde (PFA)/phosphate-buffered saline (pH 7.4). The unstained area of each slice was measured using ImageJ 1.53k software (National Institutes of Health, Bethesda, MD, USA). The sum of the TTC-unstained areas multiplied by brain thickness was calculated as the TTC-unstained volume, which corresponds to the ischemic volume [15].

### 2.3. Preparation of Poststroke Brain Samples

Poststroke mice were deeply anesthetized and transcardially perfused with 4% PFA, as described previously [14,17]. Whole brains were removed and fixed with 4% PFA. The cortical width index (CWI) was then measured by gross examination [18]. Briefly, the width was measured at the midpoint of the forebrain, and the ratio of the left cortical width (ipsilateral) excluding ischemic areas to the right cortical width (contralateral) was calculated. After brain samples were embedded in paraffin, central forebrain tissues were cut into 8 μm sections and stained with hematoxylin and eosin (H&E) or the indicated antibodies, as described previously [15].

### 2.4. Ischemic Area Evaluation

The size of the ischemic area was also estimated in poststroke neonatal and adult mouse brain sections by histopathological staining, as previously described [15]. Briefly, the poststroke “ischemic area” was measured in H&E-stained sections using ImageJ. The proportion of ischemic area (%) was calculated as follows: % ischemic area = [(contralateral hemisphere area) − (intact area of infarcted hemisphere)]/[(contralateral hemisphere area) × 2] × 100 (Supplementary Figure S1), as described previously [15].

### 2.5. Immunohistochemistry

Immunohistochemistry was performed as described [15]. In brief, paraffin-embedded 8 μm brain sections were deparaffinized, heated for 10 min in citrate buffer (pH 6.0; Abcam, Cambridge, UK) for epitope repair, and immunostained using a primary antibody against the mature neuronal marker microtubule-associated protein 2 (MAP2; 1:500, mouse, Sigma-Aldrich). Immunostained sections with 3,3′-diaminobenzide tetrahydrochloride (Vector Laboratories Inc., Burlingame, CA, USA) were counterstained with hematoxylin and photographed under a light microscope (Olympus, Tokyo, Japan) using a digital camera system.

In another set of experiments, brain sections were incubated with primary antibodies against nestin (1:100, rabbit, Abcam), glial fibrillary acidic protein (GFAP; 1:500, rabbit; Abcam), platelet-derived growth factor receptor-beta (PDGFRβ; 1:200, goat, R&D Systems, Minneapolis, MN, USA), and (or) doublecortin (DCX; 1: 1000, rabbit, Abcam). The brain sections were then incubated in Alexa Fluor 488- or 555-conjugated secondary antibodies (1:500; Molecular Probes, Eugene, OR, USA). Cell nuclei were counterstained with 4′,6-diamidino-2-phenylindole (DAPI, 1:500; Kirkegaard & Perry Laboratories, Inc., Gaithersburg, MD, USA). Stained sections were photographed using a fluorescence microscope (Olympus or LSM780, Carl Zeiss AG, Oberkochen, Germany).

The ischemic and peri-ischemic areas were defined as regions within the border of the poststroke area and within a diameter of 100 μm around the poststroke area, respectively. Areas positive for nestin and DCX and the number of cells positive for Sox2 were measured in the ischemic and peri-ischemic areas of coronal brain sections (3 regions per section from 3 mice for 9 datapoints), as described previously [15,17].

### 2.6. Cell Culture

To investigate the presence and properties of NSPCs in the nonischemic mouse brain, MCA areas (cortex) were isolated from untreated (naïve) neonatal (P13) and adult mice. To investigate the presence and properties of iNSPCs within ischemic areas, ischemic tissues were removed from neonatal (P13) and adult mice on poststroke day 7. Retrieved tissues were then dissociated and cultured under conditions that promote the formation of neurosphere-like cell clusters, as described previously [14,15,17]. Briefly, tissues were mechanically dissociated by sequential passage through 18-, 23-, and 27-gauge needles. The resulting single-cell suspension was incubated under nonadherent (floating) conditions in Dulbecco’s Modified Eagle Medium/F-12 (DMEM/F12; Thermo Fisher Scientific, Rochester, NY, USA) supplemented with 20 ng/mL basic fibroblast growth factor (bFGF; PeproTech, Rocky Hill, NJ, USA), 20 ng/mL epidermal growth factor (EGF; PeproTech), 1% N_2_ (Thermo Fisher Scientific), and 2% fetal bovine serum (FBS). Neurosphere-like cell clusters were selected and subjected to reverse transcription polymerase chain reaction (RT-PCR) (see below).

In another set of experiments, the cell clusters were differentiated in neurobasal medium (Thermo Fisher Scientific) containing bFGF, B-27 supplement (Thermo Fisher Scientific), and 2% FBS for 4 weeks, as described previously [14,15,17]. The differentiated cell clusters were analyzed by immunohistochemistry using antibodies against Tuj1 (1:1000; mouse, Stemcell Technologies, Vancouver, BC, Canada), GFAP (1:500, rabbit, Abcam), and myelin basic protein (MBP; 1:100, R&D Systems), followed by Alexa Fluor 488- or 555-conjugated secondary antibodies (1:500; Molecular Probes). The cell clusters differentiated for 2 weeks were subjected to RT-PCR (see below).

To investigate the proliferative activities of iNSPCs, neurosphere-like cell clusters were isolated and mechanically dissociated by passage through needles, as described above. The dissociated cells were cultured in poly-D-lysine-coated 6-well plates (Thermo Fisher Scientific) in DMEM/F12 (Thermo Fisher Scientific) containing bFGF (PeproTech), EGF (PeproTech), 1% N2 (Thermo Fisher Scientific), and 2% FBS by adherent culture. After reaching confluence, adhered cells were treated with trypsin and reseeded. After two passages, equal numbers of neonatal and adult iNSPCs (1 × 10^4^ cells/well) were seeded under the same conditions. Cells were trypsinized and collected after 1, 3, and 7 days. Cell numbers were measured using an automated cell counter (Cell Counter model R1, Olympus).

To investigate differences in iNSPC gene expression profiles between neonatal and adult mice, neurosphere-like cell clusters were mechanically dissociated and grown to confluence in adherent culture, as described above. After two passages, neonatal and adult iNSPCs were cultured for 7 days and treated with total RNA extraction reagents for microarray analysis. 

### 2.7. Reverse Transcription Polymerase Chain Reaction (RT-PCR)

Total RNA was extracted from differentiated and undifferentiated cell clusters derived from neonatal and adult mice using an RNeasy Micro Kit (Qiagen, Hilden, Germany), as previously described [14,17]. Target genes were quantified using the primer sequences listed in Table 1.

### 2.8. Microarray Analysis

Total RNA was extracted from iNSPCs of neonatal and adult mice as well as commercially available brain-derived pericytic cells (PCs; #M1200, ScienCell Research Laboratories, Carlsbad, CA, USA), astrocytic cells (ACs; #M1800, ScienCell Research Laboratories), endothelial cells (ECs; CRL-2299, ATCC, Manassas, VA, USA), and microglial cells (MCs; #SCC134, EMD Millipore Corporation, Temecula, CA, USA) using an RNeasy Micro Kit (Qiagen), as previously described [14,17,19]. Microarray results were analyzed using the Affymetrix Transcriptome Analysis Console [14,17,19] and Metascape gene ontology (GO) tool [20].

### 2.9. Statistical Analysis

All results are expressed as mean ± standard deviation (SD). Group means were compared using Student’s *t*-test. A *p* < 0.05 was considered statistically significant.

## 3. Results

### 3.1. Reduced Poststroke Cortical Degeneration in Neonatal Brain Compared to Adult Brain following Ischemic Stroke

**Figure 1:**
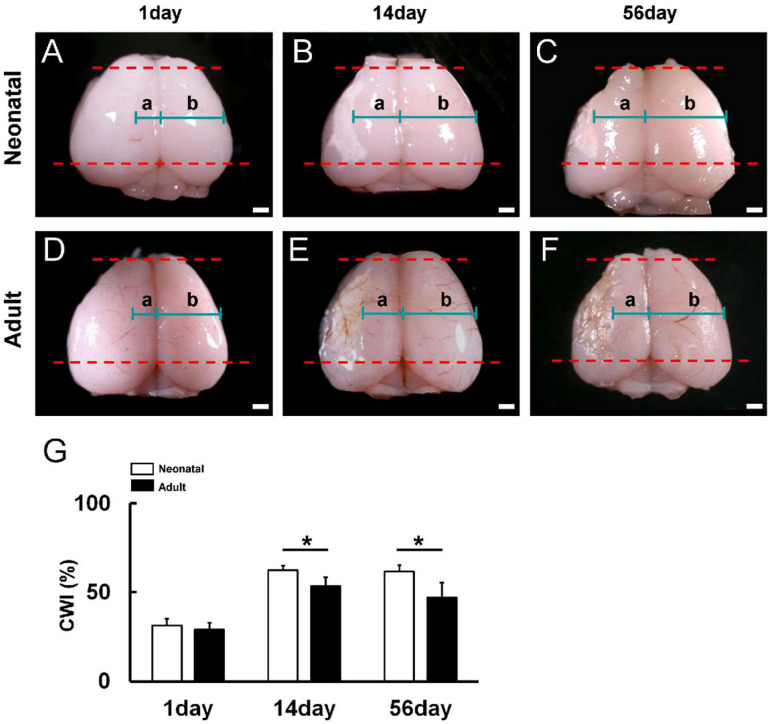
(**A**–**F**) Cortical width index (a/b ratio; a: the left cortical width (ipsilateral) excluding ischemic areas; b: the right cortical width (contralateral)) was measured at the midpoint of the forebrain (between the red dotted lines) in neonatal mice (**A**–**C**) and adult mice (**D**–**F**) at days 1 (**A**,**D**), 14 (**B**,**E**), and 56 (**C**,**F**) after MCAO. (**G**) Although the CWI was not significantly different between neonatal and adult mice on day 1 after MCAO, the CWI was significantly higher in neonatal mice than adult mice at 14 and 56 days after MCAO, indicating enhanced resistance to delayed ischemia-induced degeneration. Scale bars: 1 mm (**A**–**F**). * *p* < 0.05 between age groups by independent samples *t*-test (**G**). Results are the mean ± SD of mice per time point per group [day 1, *n* = 5 (neonatal), *n* = 3 (adult); day 14, *n* = 4 (neonatal), *n* = 7 (adult); day 56, *n* = 7 (neonatal), *n* = 3 (adult); G]. Abbreviations: CWI, cortical width index; MCAO, middle cerebral artery occlusion.

We first compared the extent and severity of ischemia-induced cortical degeneration between neonatal and adult mice by calculating the CWI (a/b ratio) on days 1, 14, and 56 after MCAO (Figure 1A–G) [18]. Although the CWI values did not differ significantly on day 1 (neonatal brains, Figure 1A,G; adult brains, Figure 1D,G), the values were significantly higher in neonatal brains on poststroke days 14 (neonatal brains, Figure 1B,G; adult brains, Figure 1E,G) and 56 (neonatal brains, Figure 1C,G; adult brains, Figure 1F,G). These results indicate that the neonatal brain has greater reparative potential and/or greater ischemic resistance than the adult brain following MCAO.

### 3.2. Reduced Ischemic Area Size in Neonatal Brain following Ischemic Stroke

**Figure 2:**
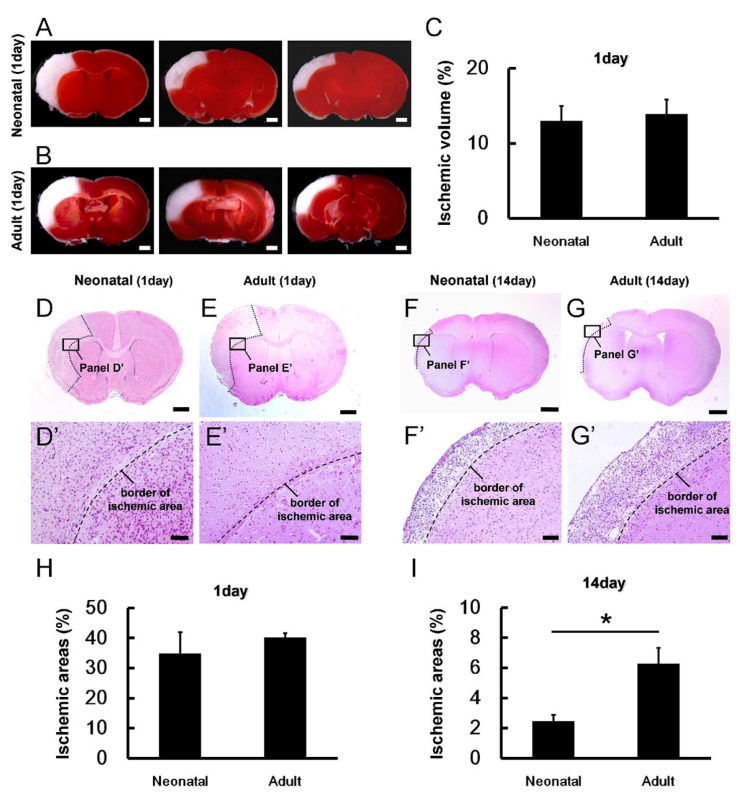
(**A**,**B**) Staining of neonatal (**A**) and adult mouse (**B**) brain slices (coronal plane) with the redox-sensitive dye TTC 1 day after MCAO. Panels A and B present different levels of the same animal. (**C**) Ischemic volume did not differ significantly between age groups during the acute phase post-MCAO. (**D**–**G**,**D’**–**G’**) H&E staining of brain sections from neonatal mice (**D**,**D’**,**F**,**F’**) and adult mice (**E**,**E’**,**G**,**G’**) revealing similar ischemic pathology on day 1 (**D**,**D’**,**E**,**E’**) but reduced ischemic injury 14 days after MCAO in neonatal brain (**F**,**F’**,**G**,**G’**). (**H**,**I**) Semiquantitative analysis indicating that the ischemic area did not differ significantly between neonatal and adult mice on day 1 after MCAO (**H**) but was significantly reduced in neonatal brain on day 14 (**I**). Scale bars: 1 mm (**A**,**B**,**D**–**G**) and 100 µm (**D’**–**G’**). * *p* < 0.05 between age groups by independent samples *t*-test (**I**). Results in C are the mean ± SD of *n* = 4 mice per group. Results in H and I are the mean ± SD of *n* = 3 mice per group. Abbreviations: H&E, hematoxylin and eosin; TTC, 2,3,5-triphenylteterazolium; MCAO, middle cerebral artery occlusion.

To compare reparative processes between neonatal and adult mice in more detail, we performed TTC and H&E staining. The staining of 2 mm coronal brain sections from neonatal mouse brain (Figure 2A) and adult mouse brain (Figure 2B) with the redox-sensitive dye TTC revealed no significant difference in relative ischemic volume (Figure 2C) on day 1 post-MCAO. However, TTC staining differentiates ischemic from nonischemic tissue only during the acute poststroke period [21,22], so we also performed H&E staining to estimate the area of ischemic damage. Consistent with the TTC staining, the ischemic area did not differ between the neonatal and adult brain on post-MCAO day 1 (neonatal brains, Figure 2D,D’,H; adult brains, Figure 2E,E’,H) but was significantly smaller in the neonatal brain on post-MCAO day 14 (neonatal brains, Figure 2F,F’,I; adult brains, Figure 2G,G’,I). 

### 3.3. Greater Neural Stem/Progenitor Cell Generation in Neonatal Brain following Ischemic Stroke

**Figure 3:**
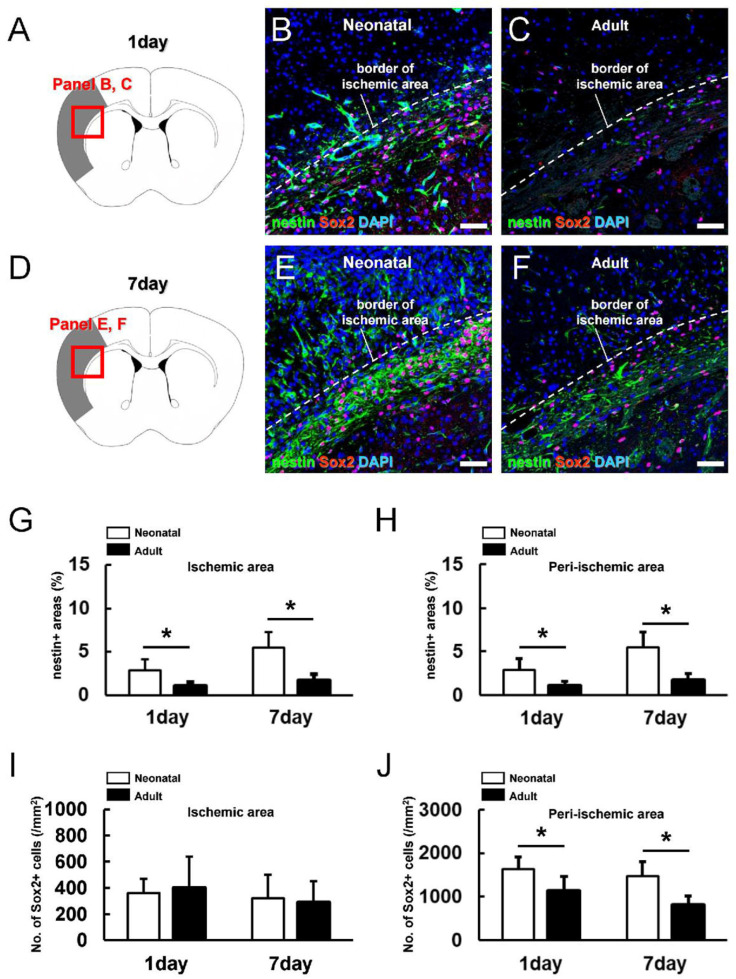
(**A**–**F**) Immunostaining for neural stem/progenitor cell (NSPC) markers nestin and Sox2 in neonatal brain (**B**,**E**) and adult brain (**C**,**F**) at 1 (**A**–**C**) and 7 days after MCAO (**D**–**F**) [nestin ((**B**,**C**,**E**,**F**): green), Sox2 ((**B**,**C**,**E**,**F**): red), DAPI ((**B**,**C**,**E**,**F**): blue)]. (**G**–**J**) The nestin^+^ areas within the ischemic (**G**) and peri-ischemic areas (**H**) were significantly larger in neonatal mice than in adult mice at 1 and 7 days after MCAO. The number of Sox2^+^ cells within ischemic areas did not differ between neonatal and adult mice at 1 and 7 days after MCAO (**I**) but was greater in the peri-ischemic areas of neonatal mice on days 1 and 7 (**J**). Scale bars = 50 µm (**B**,**C**,**E**,**F**). * *p* < 0.05 between age groups by independent samples *t*-test (**G**,**H**,**J**). In (**G**–**J**), results are the average ± SD of *n* = 3 mice per time point per age group. Abbreviations: DAPI, 4′,6-diamidino-2-phenylindole; MCAO, middle cerebral artery occlusion.

To investigate the potential mechanisms for the enhanced repair of neonatal brain following MCAO, we first estimated the rate of neuronal differentiation at sites of injury by immunostaining for the mature neuronal marker MAP2. On post-MCAO day 1, no MAP2^+^ cells were observed within the ischemic areas of the neonatal and adult brain (Supplementary Figure S2), suggesting that most neurons in these regions are destroyed or severely injured regardless of age. These findings further suggest that the reduced susceptibility of the neonatal brain to ischemic damage results from more rapid and extensive regeneration (i.e., greater reparative potential rather than greater ischemic resistance at the neuronal level). In support of this idea, immunohistochemistry (Figure 3A–F) revealed greater numbers of cells expressing the NSPC markers nestin and Sox2 within and around the ischemic areas of the neonatal brain (Figure 3B,E) compared to the adult brain (Figure 3C,F) on post-MCAO day 1 (Figure 3A–C) and day 7 (Figure 3D–F). In accord with enhanced reparative capacity in the neonatal brain, semiquantitative analysis revealed significantly larger nestin^+^ areas within (Figure 3G) and around the ischemic regions (Figure 3H) of the neonatal brain compared to the adult brain on post-MCAO days 1 and 7. And also, although Sox2^+^ cells within ischemic areas did not differ by age (Figure 3I), Sox2^+^ cells were more numerous around ischemic areas on post-MCAO days 1 and 7 (Figure 3J).

**Figure 4:**
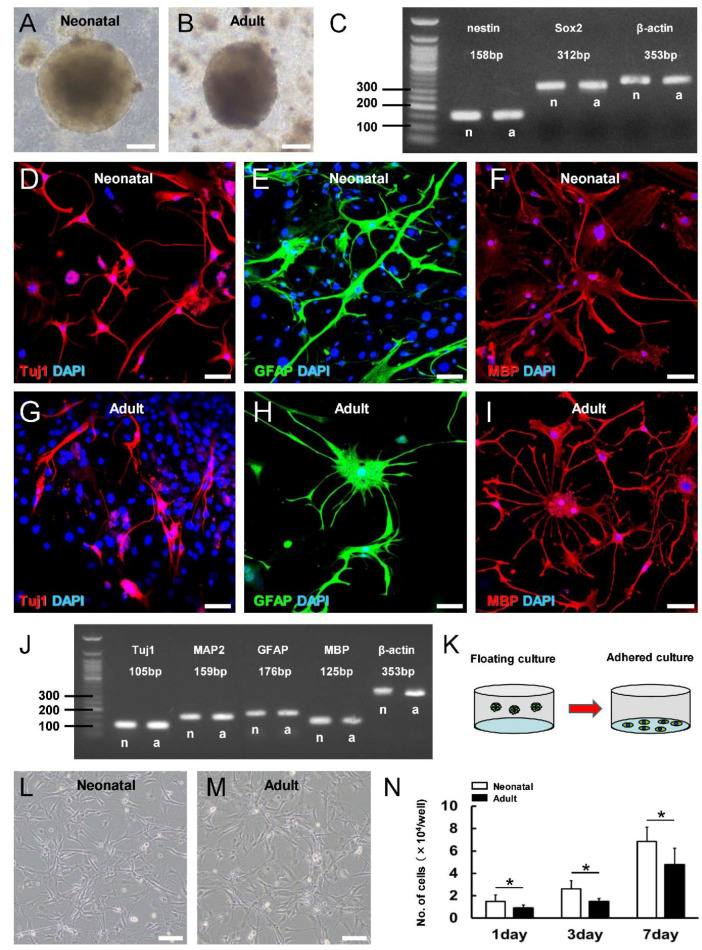
(**A**,**B**) Neurosphere-like cell clusters were obtained from neonatal (**A**) and adult mice (**B**) after ischemic stroke. (**C**) RT-PCR confirmed that the cell clusters from neonatal (n) and adult mice (a) expressed the NSPC markers nestin and Sox2. (**D**–**I**) Immunostaining showing that cells from neonatal mice (**D**–**F**) and adult mice (**G**–**I**) can differentiate into cells expressing the neuronal marker Tuj1 ((**D**,**G**): red), the astrocytic marker GFAP ((**E**,**H**): green), and the oligodendrocyte marker MBP ((**F**,**I**): red). Nuclei were counterstained with DAPI ((**D**–**I**): blue). (**J**) RT-PCR analysis confirming that differentiated cells from neonatal (n) and adult mice (a) express marker genes of mature neurons (Tuj1, MAP2), astrocytes (GFAP), and oligodendrocytes (MBP). (**K**–**M**) Stem cells from neonatal cortex proliferate faster in adherent culture. Neurosphere-like cell clusters obtained from both neonatal (**L**) and adult brain (**M**) were reseeded in adherent cultures. (**N**) Cell number was significantly higher in adherent cultures derived from neonatal mouse neurospheres than adult mouse neurospheres at 1, 3, and 7 days postseeding. Scale bars = 100 µm (**A**,**B**,**L**,**M**) and 50 µm (**D**–**I**). * *p* < 0.05 between age groups by independent samples *t*-test (**N**). Results are the mean ± SD of 3 samples of *n* = 3 mice (9 date points) per time point per group (**N**). Abbreviations: DAPI, 4′,6-diamidino-2-phenylindole; GFAP, glial fibrillary acidic protein; MAP2, microtubule-associated protein 2; MBP, myelin basic protein; MCAO, middle cerebral artery occlusion.

To examine if this greater iNSPC response in the neonatal brain results in higher basal activity or more robust induction by ischemia, NSPCs were isolated from the MCA fields of naïve and ischemia-treated neonatal (P13) and adult mice and compared for growth and differentiation potential in vitro. Neurosphere-like cell clusters were not observed in floating cultures from either naïve neonatal or adult brain, even after 4 weeks, indicating that there are few if any NSPCs in MCA areas under physiological conditions. Following MCAO, however, neurosphere-like cell clusters were observed in cultures from both the neonatal (Figure 4A) and adult brain (Figure 4B). Moreover, RT-PCR showed that the neurosphere-like cell clusters from both age groups expressed the NSPC markers nestin and Sox2 (Figure 4C). After further culture under differentiation conditions, neurosphere-like cell clusters from neonatal (Figure 4D–F) and adult mice (Figure 4G–I) expressed the neuronal marker Tuj1 (Figure 4D,G), astrocytic marker GFAP (Figure 4E,H), and oligodendrocyte marker MBP (Figure 4F,I). These results were further confirmed by gene expression analysis using RT-PCR (Figure 4J). Collectively, these results strongly suggest that iNSPCs which have the potential to differentiate into neurons, astrocytes, and oligodendrocytes are induced in both the neonatal and adult brain following ischemia. However, this process appears to be more efficient in the neonatal brain, as evidenced by the histopathological and immunohistochemical staining results.

To examine if this more robust iNSPC response results from greater proliferative potential, we compared proliferation rates between ischemia-exposed neurosphere-derived cells from neonatal and adult mice in adherent culture (Figure 4K). Indeed, neurosphere-derived single-cell suspensions from both neonatal (Figure 4L) and adult mice (Figure 4M) yielded proliferating cells that reached confluence in adherent culture. However, suspensions from neonatal mice yielded greater numbers of iNSPCs after 1, 3, and 7 days of adherent culture (Figure 4N). These differences in the proliferative potential of iNSPCs between neonatal and adult mice may contribute to the higher numbers of iNSPCs observed in the neonatal brain after ischemic stroke (Figure 3).

### 3.4. Characterization of iNSPC Lineages and Phenotypes in Neonatal Brain after Ischemic Stroke

**Figure 5:**
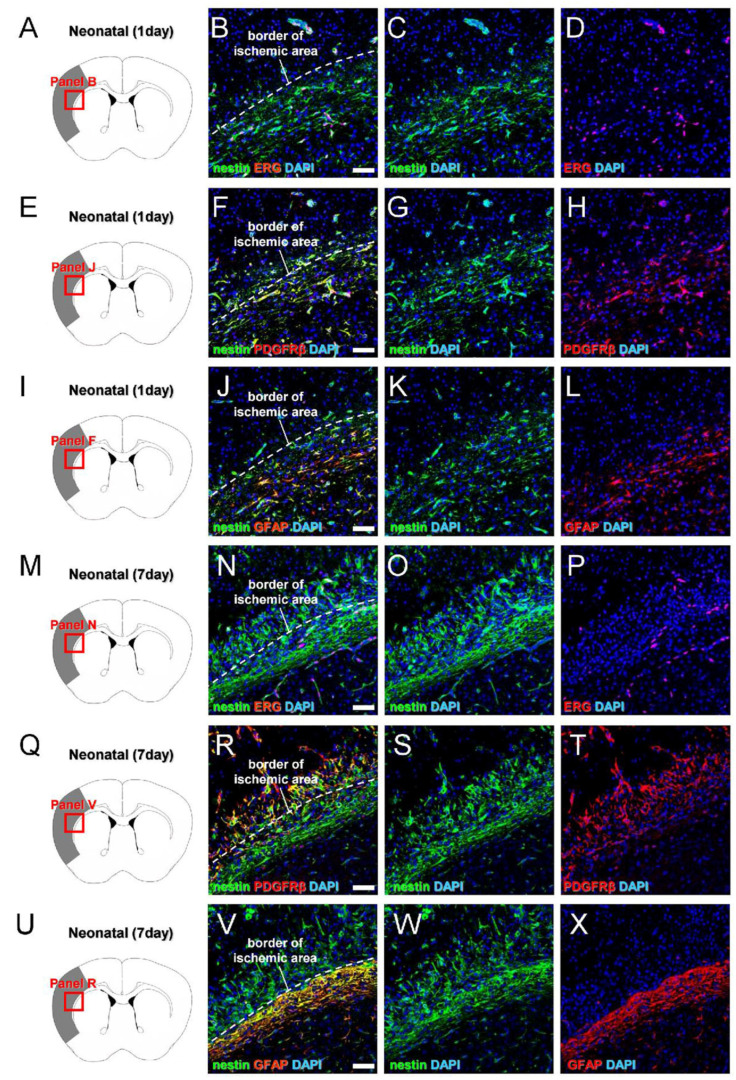
(**A**–**X**) Immunostaining for nestin and ERG (**A**–**D**,**M**–**P**), nestin and PDGFRβ (**E**–**H**,**Q**–**T**), and nestin and GFAP (**I**–**L**,**U**–**X**) in neonatal mouse brain sections isolated at 1 (**A**–**L**) and 7 days after MCAO (**M**–**X**) [nestin ((**B**,**C**,**F**,**G**,**J**,**K**,**N**,**O**,**R**,**S**,**V**,**W**): green), ERG ((**B**,**D**,**N**,**P**): red), PDGFRβ ((**F**,**H**,**R**,**T**): red), GFAP ((**J**,**L**,**V**,**X**): red), DAPI ((**B**–**D**,**F**–**H**,**J**–**L**,**N**–**P**,**R**–**T**,**V**–**X**): blue)]. Scale bars = 50 µm (**B**,**F**,**J**,**N**,**R**,**V**). Abbreviations: DAPI, 4′,6-diamidino-2-phenylindole; ERG, ETS-related gene; GFAP, glial fibrillary acidic protein; MCAO, middle cerebral artery occlusion; PDGFRB, platelet-derived growth factor receptor-beta.

We then examined the precise origins and traits of iNSPCs in the neonatal brain by marker expression analyses. Previous studies have shown that iNSPCs in the adult brain originate from multiple cell types, including reactive pericytes [23] and reactive astrocytes [24,25], so we first examined the coexpression of nestin with pericyte and astrocyte markers on post-MCAO day 1 (Figure 5A–L) and day 7 (Figure 5M–X). On day 1, nestin^+^ cells were largely localized around ERG^+^ endothelial cells (Figure 5A–D), and most were positive for the pericyte marker PDGFRβ (Figure 5E–H), whereas some in peri-ischemic areas expressed the astrocytic marker GFAP (Figure 5I–L). On day 7, some nestin^+^ cells were located around ERG^+^ cells (Figure 5M–P). However, most nestin^+^ cells within ischemic areas expressed PDGFRβ (Figure 5Q–T), whereas nestin^+^ cells in peri-ischemic areas expressed GFAP (Figure 5U–X). These results suggest that most nestin^+^ cells within the ischemic areas of the neonatal brain originate from pericytes rather than reactive astrocytes, which is consistent with previous studies of iNSPCs in adult ischemic brain [23].

**Figure 6:**
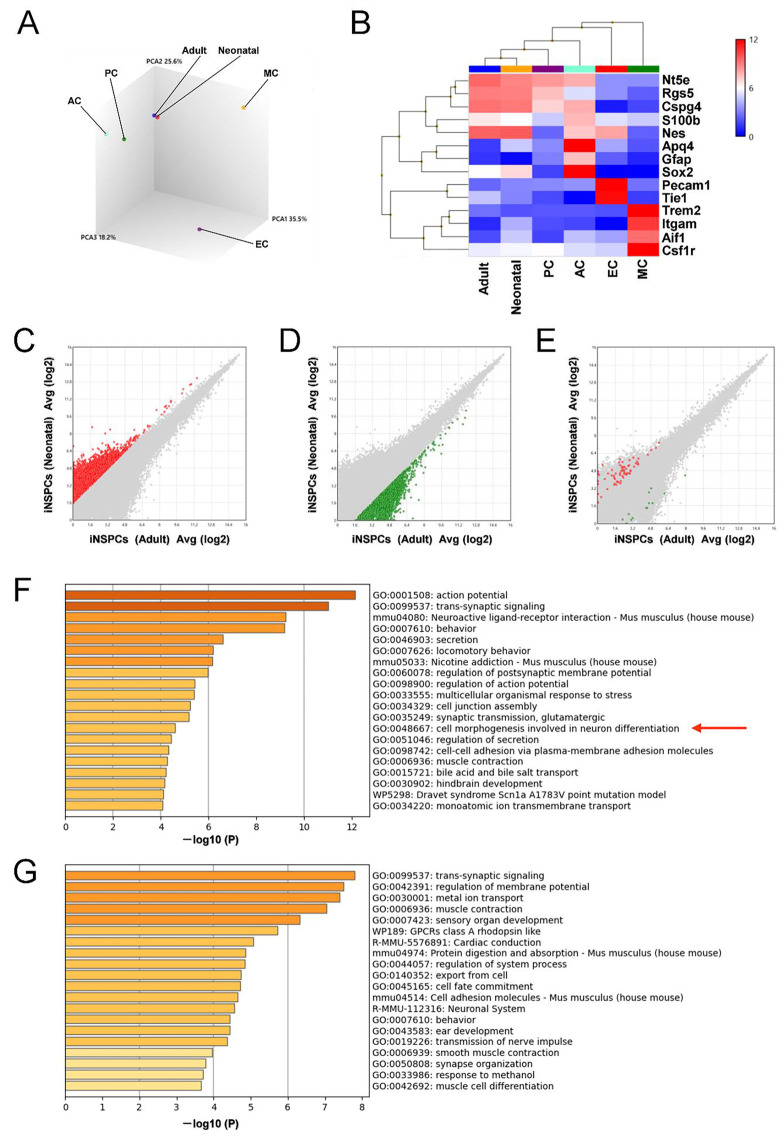
(**A**,**B**) Principal component analysis (PCA) (**A**) and heatmap (**B**) for the gene expression profiles of neonatal iNSPCs, adult iNSPCs, pericytic cells (PCs), astrocytes (ACs), endothelial cells (ECs), and microglial cells (MCs). (**C**–**E**) Scatter plots showing the distribution of genes upregulated more than 3-fold in iNSPCs from neonatal mice compared to adult mice (**C**, red plots) or genes upregulated more than 3-fold in iNSPCs from adult mice compared to neonatal mice (**D**, green plots). (**E**) The scatter plot analysis shows the distribution of genes categorized in the “cell morphogenesis involved in neuron differentiation” category ((a red arrow in (**F**)). (**F**,**G**) List of the top 20 categories for genes overexpressed in neonatal iNSPCs (**F**) and adult iNSPCs (**G**) by GO analysis. Abbreviations: AC, astrocyte; EC, endothelial cell; GO, gene ontology; iNSPC, injury/ischemia-induced neural stem/progenitor cell; MC, microglial cell; PC, pericyte; PCA, principal component analysis.

Microarray analysis was then performed to investigate iNSPC phenotype in greater detail. Principal component analysis (PCA) indicated that the gene expression profiles of iNSPCs from neonatal mice were closer to those from adult mice than pericytes (PCs), astrocytes (ACs), endothelial cells (ECs), and microglial cells (MCs) (Figure 6A). Heatmap analysis indicated that the gene expression profiles of iNSPCs from both neonatal and adult mice were closer to PCs (markers CSPG4, RGS5, NT5E) than to ACs (GFAP, AQP4, S100B), ECs (PECAM1, TIE1), and MCs (AIF1, CSF1R, ITGAM, TREM2) (Figure 6B).

We further investigated the functions of differentially expressed genes (DEGs, defined as > 3-fold greater expression in neonatal brain than adult brain; red dots in Figure 6C) by gene chip and GO analyses. The top 20 GO categories related to nervous system included “GO:0001508: action potential”, “GO:0099537: trans-synaptic signaling”, “mmu04080: neuroactive ligand–receptor interaction”, “GO:0060078: regulation of postsynaptic membrane potential”, “GO:0098900: regulation of action potential”, “GO:0035249: synaptic transmission, glutamatergic”, and “GO:0048667: cell morphogenesis involved in neuron differentiation” (Figure 6F), whereas similar GO analysis of upregulated DEGs in adult iNSPCs (defined as > 3-fold greater expression in adult brain than neonatal brain; green dots; Figure 6D) yielded “GO:0099537: trans-synaptic signaling”, “R-MMU-112316: neuronal system”, “GO:0019226: transmission of nerve impulse”, and “GO:0050808: synapse organization”.

Based on the inclusion of the GO term “GO:0048667: cell morphogenesis involved in neuron differentiation”, we further investigated the distribution of genes in this category in red (Figure 6C) and green dots (Figure 6D) by scatter plot analysis. Although certain genes included in this category (Supplementary Table S1) were upregulated in either the neonatal or adult brain (red or green dots, respectively), a greater number of genes were upregulated in the neonatal brain (Figure 6E), suggesting that iNSPCs in the neonatal brain possess a greater potential to differentiate into neuronal cells than those from adult mice.

### 3.5. iNSPCs from Neonatal Brain Show a Greater Potential for Neurogenesis after Ischemic Stroke

**Figure 7:**
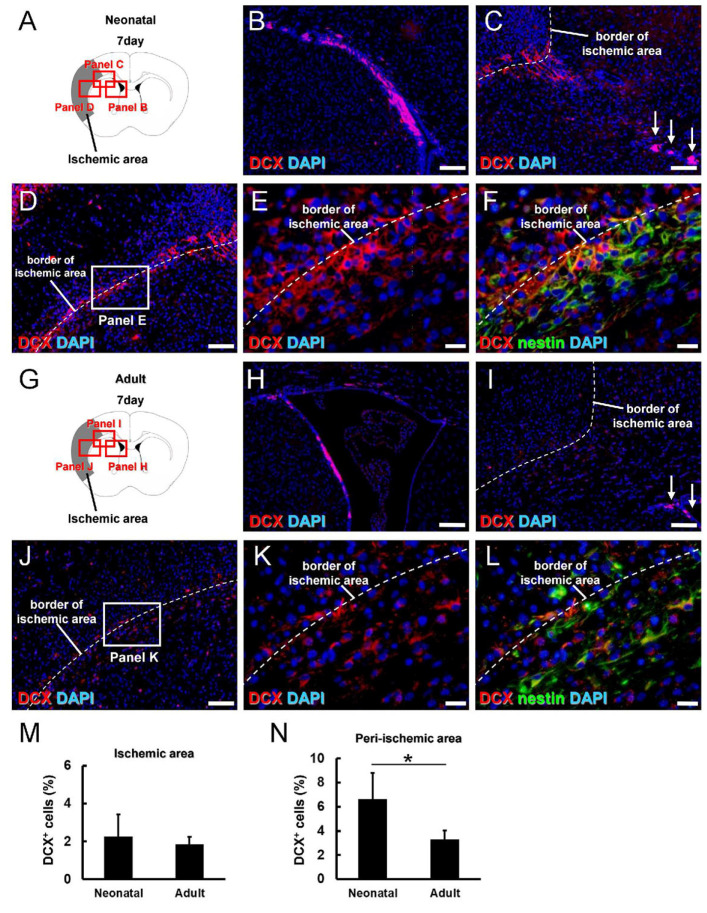
(**A**–**L**) Immunostaining for the newborn neuron marker doublecortin (DCX, (**A**–**L**)) and nestin (**F**,**L**) in brain sections from neonatal (**A**–**F**) and adult mice (**G**–**L**) at 7 days after MCAO [DCX ((**B**–**F**,**H**–**L**): red), nestin ((**F**,**L**): green), DAPI ((**B**–**F**,**H**–**L**): blue)]. DCX^+^ cells were observed in the subventricular zone (SVZ) of neonatal mice (**B**,**C**) and adult mice (**H**,**I**), but these cells did not reach ischemic areas ((**C**,**I**); arrows). DCX^+^ cells were also observed within ischemic and peri-ischemic areas of neonatal (**C**–**F**) and adult cortex (**I**–**L**) and some coexpressed nestin (**F**,**L**). (**M**,**N**) Quantitative analysis showing that the DCX^+^ areas within ischemic areas did not differ between neonatal and adult mice at 7 days after MCAO (**M**) but were significantly higher in peri-ischemic areas of neonatal mice (**N**). Scale bars = 100 µm (**B**–**D**,**H**–**J**) and 20 µm (**E**,**F**,**K**,**L**). * *p* < 0.05 between age groups by independent samples *t*-test (**N**). Results in M and N are the mean ± SD of *n* = 3 mice per group. Abbreviations: DAPI, 4′,6-diamidino-2-phenylindole; MCAO, middle cerebral artery occlusion.

To directly compare the neurogenic potential of iNSPCs from the neonatal and adult brain after ischemic stroke, we investigated the expression patterns of doublecortin (DCX) (Figure 7A). On day 7 post-MCAO, many DCX^+^ cells were observed in the contralateral SVZ (Supplementary Figure S3A,B) but rarely in the contralateral nonischemic areas (Supplementary Figure S3A,C,D). Immunohistochemistry at post-MCAO day 7 also revealed many DCX^+^ cells in the ipsilateral SVZ (Figure 7B) as well as both within and around ischemic areas (Figure 7C–E). Notably, however, DCX^+^ cells in the SVZ did not reach into ischemic areas (Figure 7C, arrows). In addition, double immunohistochemistry for DCX and nestin showed that many DCX^+^ cells were localized near nestin^+^ cells and that some coexpressed nestin (Figure 7F). Taken together, these findings suggest that locally activated nestin^+^ iNSPCs generate DCX^+^ cells following ischemia, in accord with previous studies [26,27].

Next, the expression patterns of DCX^+^ cells were investigated in adult mice (Figure 7G–L). On post-MCAO day 7, some DCX^+^ cells were detected in the contralateral SVZ (Supplementary Figure S3E,F) but rarely in the contralateral nonischemic areas (Supplementary Figure S3E,G,H). Alternatively, numerous DCX^+^ cells were observed at the ipsilateral SVZ (Figure 7H) and within and around ischemic areas (Figure 7I,J,K). A similar pattern of nestin^+^/DCX^+^ cells was detected within and around the ischemic areas of adult mice (Figure 7L), again suggesting the activation of a local population. While the DCX^+^ areas within ischemic areas did not differ between neonatal and adult brains on day 7 (Figure 7M), the DCX^+^ areas in peri-ischemic areas were significantly higher in the neonatal brain (Figure 7N), consistent with greater neurogenic potential.

## 4. Discussion

This is the first study to compare reparative processes after experimental ischemic stroke between neonatal (P13) and adult mice (8–12 weeks old). We showed that the lower susceptibility of neonatal cortex to ischemic damage is conferred by a greater capacity to produce iNSPCs and newborn neurons in the peri-ischemic area. Although the precise relationships between mouse and human developmental stages are still debated [28], P0 to P28 is considered to be within the neonatal stage in mice [29], whereas 8 weeks and older is generally regarded as adulthood based on the emergence of reproductive and territorial behaviors. Thus, younger patients may exhibit greater regenerative activities than older patients through a similar mechanism observed in the current study using neonatal (P13) and adult mice (8–12 weeks old) even after brain injury. However, the brains of mice rapidly develop within a shorter span than those of humans. Therefore, studies on animals with a slower development (e.g., the naked mole-rat) [30] may provide comparable insights on reparative processes at different ages in pathological brain conditions. 

Ischemic stroke is both less frequent and less deadly during the neonatal period than during late adulthood. While seizure is a common sequela of neonatal stroke [4], more severe complication such as paresis are less frequent than following adult stroke [8,9], resulting in superior short-term prognosis [10]. In addition, long-term follow-up studies have reported normal neurological scores at preschool age among children with a history of neonatal stroke, although IQ scores are lower than expected by school age [10]. The mouse stroke model used in the current study has demonstrated reproducibly high survival rates [14,15] and so may be useful for evaluating these delayed cognitive sequela [14,15] and the underlying mechanisms.

The present results suggest that greater neural reparative capacity may explain this superior clinical outcome following neonatal stroke. Neonatal mice demonstrated significantly greater iNSPC generation capacity compared to adult mice as well as greater neurogenic potential, possibly due to the upregulation of genes related to “cell morphogenesis involved in neuron differentiation”. However, this greater neurogenic capacity may also contribute to undesirable complications (e.g., seizure). In support of this idea, GO analysis revealed that genes upregulated in neonatal mice following MCAO were enriched in the functional annotations “action potential” and “regulation of action potential”.

During the early embryonic stage, neurogenesis is detectable throughout the brain, including in the cortex. Mignone and colleagues [12] reported that GFP expression driven by the nestin promoter was first detectable on embryonic day 7 (E7), observed mainly in the neural plate at E8 and then distributed throughout the neuroepithelium by E10, but was largely restricted to specific regions such as ventricular zones by E12 and finally detectable only in conventional neurogenic regions such as the SVZ and SGZ by adulthood. Thus, NSPC activities, including NSPC-dependent neurogenesis, likely also decrease gradually with brain development outside regions such as the SVZ and SGZ [13,31]. In support of this notion, no neurosphere-like cell clusters were obtained from MCA areas of naïve neonatal cortex (P13). However, previous studies have shown that neurogenesis can be reactivated in the adult brain outside of conventional neurogenic zones under pathological conditions [32,33,34]. For instance, we previously demonstrated that nestin^+^ iNSPCs could be obtained from MCA areas of adult mouse cortex following ischemic stroke [14,15]. In the present study as well, we report that iNSPCs were activated in large numbers by cortical ischemia in both the neonatal and adult cortex. However, the areas and number of cells expressing the NSPC markers nestin and Sox2 were significantly higher in the neonatal brain compared to the adult brain, possibly due to a high proliferation rate, as suggested by in vitro assays. 

Alternatively, iNSPCs in the neonatal cortex may be more efficiently induced by ischemia or related factors. For example, the development of extracellular matrix, which is closely associated with NSPC regulation [35,36], differs between neonatal and adult mice [37]. In addition, vascular development differs between neonatal and adult mice [37,38,39,40]. We previously showed that some vascular lineage cells can survive even after ischemic insult [15], and vascular lineage cells (e.g., ECs) can serve as stem cell niches for iNSPCs within and around ischemic areas [41]. Thus, such differences between developmental stages may affect the fate of iNSPCs. 

In addition, environmental conditions around stem cells (e.g., stem cell niches) are dramatically altered at sites of injury after ischemic stroke, and we previously reported that inflammatory cells [e.g., T lymphocytes [42] and microglial cells/macrophages [17]] serve as stem cell niches for iNSPCs within and around the ischemic areas and influence the fate of these iNSPCs. Furthermore, the inflammatory response after spinal cord injury differs between developing and mature rats [43]. Thus, it is possible that the responsivity of iNSPCs to various stem cell niches differs between the neonatal and adult brain under pathological conditions. Factors regulating iNSPC fate may be effective tools to enhance neural regeneration after brain injuries.

The precise origins of iNSPCs in the neonatal brain remain unclear. We found that nestin^+^ cells within ischemic areas mainly expressed PDGFRβ, whereas nestin^+^ cells in peri-ischemic areas mainly expressed GFAP, suggesting distinct pericyte and astroglial lineages. We have also found stem cells that likely originate from brain pericytes which are neural crest derivatives [44], with the capacity to differentiate along multiple lineage pathways, both in adult mouse brains subjected to ischemic stroke [23] and human brain samples from patients with stroke [19]. In addition, we previously reported that nestin^+^/PDGFRβ^+^ brain pericytes extracted from ischemic areas of adult mouse brain converted to nestin^+^/Sox2^+^ iNSPC-like cells via a mesenchymal–epithelial transition mechanism [19]. Nestin^+^/Sox2^+^ cells were predominantly localized in peri-ischemic areas of the neonatal brain, suggesting that nestin^+^ cells in peri-ischemic areas may have higher iNSPC activity than nestin^+^ cells in the ischemic core. However, peri-ischemic areas may include the corpus callosum, a region prolific with oligodendrocyte precursor cells, which can transform into NSPCs [45,46]. Thus, the precise traits and origin of iNSPCs in peri-ischemic areas should be elucidated in future studies.

We also found a significantly greater generation of DCX^+^ (newborn) neurons in the neonatal brain than in the adult brain, consistent with greater neurogenic potential. However, this elevation was restricted to peri-ischemic areas, suggesting that the microenvironmental conditions in ischemic areas may be hostile to iNSPC differentiation into neurons. In support of this idea, we previously demonstrated that iNSPCs can differentiate into neuronal cells if transplanted into peri-ischemic areas of mouse brain but not if transplanted within ischemic areas [41]. Similarly, differentiation into neural lineages may be higher in peri-ischemic areas than in ischemic areas of the neonatal brain, as evidenced by the predominant localization of nestin^+^/Sox2^+^ cells in peri-ischemic areas. Some DCX^+^ cells in and around ischemic areas also expressed nestin but formed a population separate from DCX^+^ cells in the SVZ. These findings suggest that DCX^+^ cells in ischemic areas are not likely SVZ-derived but rather arise from regionally activated stem cells. In support of this idea, we and others have reported that nestin^+^/DCX^+^ cells are induced in regions far away from the SVZ, such as leptomeninges, in the adult mouse brain following brain and spinal cord injuries [26,27]. In addition, using transgenic mice that can trace the fates of nestin^+^ endogenous NSPCs, we demonstrated that iNSPCs that occur within and around ischemic areas during the acute period are derived from cells in situ but not from SVZ-derived NSPCs [14]. Although the migratory capacity of SVZ-derived NSPCs is limited during the acute period [47,48], DCX^+^ cells in the SVZ can migrate around the injured areas during the chronic period after MCAO [49]. Therefore, the precise origins and traits of iNSPCs and newborn neurons in the neonatal brain should be clarified in future studies.

Our study has several limitations. For example, long-term follow-up studies to determine the causes of the elevated neural reparative activity in neonatal brains should be investigated. Determining if the larger cortical areas (greater CWI) in neonatal brains are primarily due to neurogenesis, gliogenesis, or both is also important. In addition, differences in reparative vasculogenesis processes after ischemic stroke between neonatal and adult brains should be investigated. If significant neurological functional recovery is observed in neonatal brains compared with adult brains, the cause of the reparative processes should be determined (neurogenesis, gliogenesis, and/or vasculogenesis).

In conclusion, we show that neural reparative potential is substantially higher in neonatal brains than in adult brains after ischemic stroke due in part to greater iNSPC expansion and neurogenic capacities. These findings not only reveal novel aspects of ischemic stroke pathogenesis but also identify potential targets to enhance iNSPC-mediated neuronal repair as a treatment for degenerative brain disorders.

## Figures and Tables

**Table 1:** List and sequences of mouse primers used for RT-PCR analysis.

Primers	Sequence (5′→3′) (F: Forward; R: Reverse)	Size
β-actin	F: GCTCGTCGTCGACAAGGGCTC R: CAAACATGATCTGGGTCATCTTCTC	353 bp
GFAP	F: TCGGCCAGTTACCAGGAGG R: ATGGTGATGCGGTTTTCTTCG	176 bp
MAP2	F: CTCATTCGCTGAGCCTTTAGAC R: ACTGGAGGCAACTTTTCTCCT	159 bp
MBP	F: TCACAGCGATCCAAGTACCTG R: CCCCTGTCACCGCTAAAGAA	125 bp
nestin	F: CGCTGGAACAGAGATTGGAAG R: CATCTTGAGGTGTGCCAGTT	158 bp
Sox2	F: TTGGGAGGGGTGCAAAAAGA R: CCTGCGAAGCGCCTAACGTA	312 bp
Tuj1	F: TGAGGCCTCCTCTCACAAGT R: GGCCTGAATAGGTGTCCAAA	105 bp

## Data Availability

The data supporting this article will be shared by the corresponding author upon reasonable request.

## Supplementary Materials

The following supporting information can be downloaded at https://www.mdpi.com/article/10.3390/cells13060519/s1, Figure S1: Schematic representation of the formula for calculating “% ischemic area”. Area “A” represents contralateral hemisphere area (marked in red and demarcated by a red line). Area “B” represents the intact area of the infarcted hemisphere (marked in blue and demarcated by a blue line). % ischemic area = [(contralateral hemisphere area) − (intact area of infarcted hemisphere)]/[(contralateral hemisphere area) × 2] × 100; in other words, % ischemic area = [A − B]/[A × 2] × 100; Figure S2: Immunohistochemistry of MAP2 in the brain sections from neonatal (A,A’,A’’) and adult mice (B,B’,B’’) on day 1 after MCAO. Scale bars = 1 mm (A,B) and 50 μm (A’,A’’,B’,B’’). Abbreviations: MAP2, microtubule-associated protein 2; MCAO, middle cerebral artery occlusion; Figure S3: Immunohistochemistry of DCX in the contralateral brain sections from neonatal (A–D) and adult mice (E–H) at 7 days after MCAO [DCX (B–D,F–H: red), DAPI (B–D,F–H: blue)]. DCX^+^ cells were restricted within the SVZ in neonatal (B) and adult mice (F). Scale bars = 100 μm (B–D,F–H). Abbreviations: DAPI, 4′,6-diamidino-2-phenylindole; DCX, doublecortin; MCAO, middle cerebral artery occlusion; SVZ, subventricular zone. Table S1: The genes included in “GO:0048667:cell morphogenesis involved in neuron differentiation” and the values of fold change (iNSPCs from neonatal mice relative to iNSPCs from adult mice).
