# Supplementary material for: Neonatal Brains Exhibit Higher Neural Reparative Activities than Adult Brains in a Mouse Model of Ischemic Stroke
Source: Cells. 2024 Mar 15;13(6):519. doi: 10.3390/cells13060519 (PMC10969155; doi:10.3390/cells13060519)
Supplement: Supplementary file 1 [file cells-13-00519-s001.zip › cells-2851021-supplementary.pdf]

Supplementary Figure S1

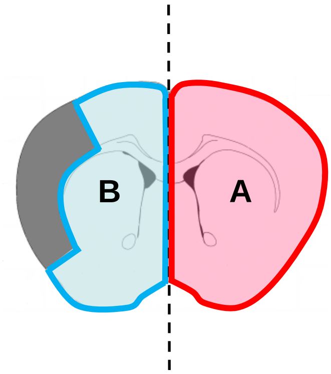

Midline of hemisphere

Area "A": Contralateral hemisphere area
(marked in red and demarcated by a red line)

Area "B": Intact area of the infarcted hemisphere
(marked in blue and demarcated by a blue line)

Supplementary Figure S2

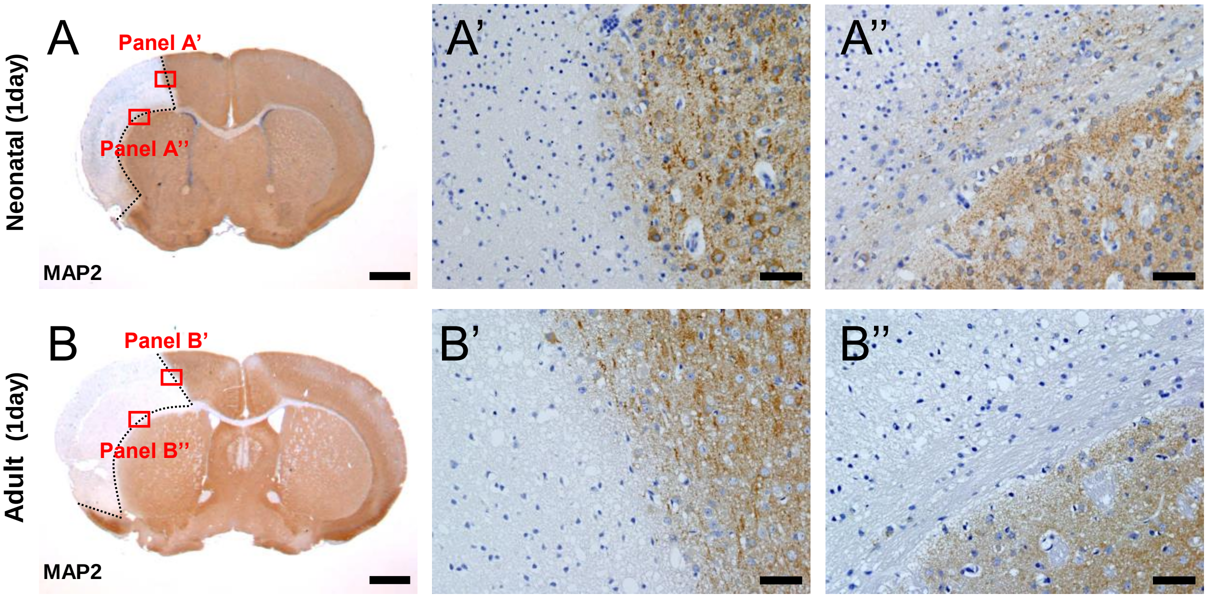

Supplementary Figure S3

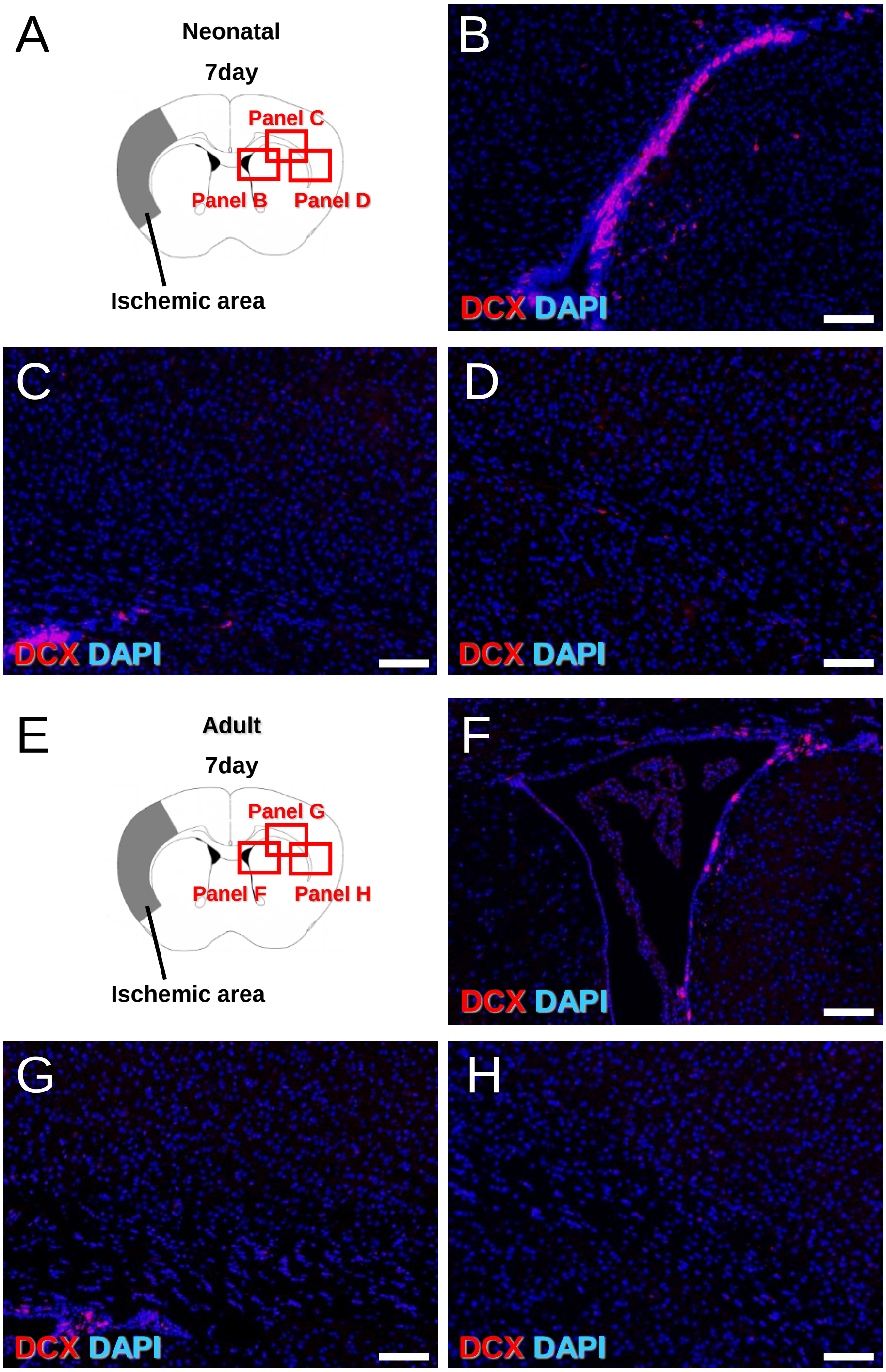

Supplementary Figure legends

Figure S1

Schematic representation of the formula for calculating "% ischemic area". Area "A" represents contralateral hemisphere area (marked in red and demarcated by a red line). Area "B" represents the intact area of the infarcted hemisphere (marked in blue and demarcated by a blue line). % ischemic area = [(contralateral hemisphere area) − (intact area of infarcted hemisphere)]/[(contralateral hemisphere area) × 2] × 100; in other words, % ischemic area = [A − B]/[A × 2] × 100.

Figure S2

Immunohistochemistry of MAP2 in the brain sections from neonatal (A, A', A'') and adult mice (B, B', B'') on day 1 after MCAO. Scale bars = 1 mm (A, B) and 50 μm (A', A'', B', B''). Abbreviations: MAP2, microtubule-associated protein 2; MCAO, middle cerebral artery occlusion.

Figure S3

Immunohistochemistry of DCX in the contralateral brain sections from neonatal (A–D) and adult mice (E–H) at 7 days after MCAO [DCX (B–D, F–H: red), DAPI (B–D, F–H: blue)]. DCX^+ cells were restricted within the SVZ in neonatal (B) and adult mice (F). Scale bars = 100 μm (B–D, F–H). Abbreviations: DAPI, 4',6-diamidino-2-phenylindole; DCX, doublecortin; MCAO, middle cerebral artery occlusion; SVZ, subventricular zone.

Supplementary Table S1

The genes included in "GO:0048667: cell morphogenesis involved in neuron differentiation" and the values of fold change (iNSPCs from neonatal mice relative to iNSPCs from adult mice)

Gene symbol	Gene name	ID	Fold change
Cntn1	contactin 1	1449563_at	29.91
Ablim1	actin-binding LIM protein 1	1442376_at	26.71
Ank3	ankyrin 3, epithelial	1452872_at	23.12
Dcdc2a	doublecortin domain containing 2a	1459661_at	19.86
Slc1a3	solute carrier family 1 (glial high affinity glutamate transporter), member 3	1440491_at	14.54
Lhx3	LIM homeobox protein 3	1425041_at	14.47
Megf9	multiple EGF-like-domains 9	1455960_at	12.58
Lpar3	lysophosphatidic acid receptor 3	1418723_at	12.31
Lgi1	leucine-rich repeat LGI family, member 1	1435851_at	12.13
Elavl4	ELAV (embryonic lethal, abnormal vision, Drosophila)-like 4 (Hu antigen D)	1452894_at	12.12
Itgb1	integrin beta 1 (fibronectin receptor beta)	1438119_at	10.58
Pcdh15	protocadherin 15	1421503_at	10.29
Pax6	paired box 6	1419271_at	9.9
Ptprz1	protein tyrosine phosphatase, receptor type Z, polypeptide 1	1427019_at	8.7
Myo5b	myosin VB	1441104_at	8
Cckar	cholecystokinin A receptor	1421195_at	7.64
Actl9	actin-like 9	1437038_x_at	7.53
Cacna1a	calcium channel, voltage-dependent, P/Q type, alpha 1A subunit	1430408_at	7.3
Kalrn	kalirin, RhoGEF kinase	1448023_at	7.1
Dscam	Down syndrome cell adhesion molecule	1419293_at	6.88
Sult4a1	sulfotransferase family 4A, member 1	1421606_a_at	6.87
Epha3	Eph receptor A3	1426057_a_at	6.52
Dscaml1	Down syndrome cell adhesion molecule like 1	1441706_at	6.51
Zeb2	zinc finger E-box binding homeobox 2	1454200_at	6.43
Zdhhc17	zinc finger, DHHC domain containing 17	1458363_at	6.29

Isl1	ISL1 transcription factor, LIM/homeodomain	1450723_at	5.97
Tenm2	teneurin transmembrane protein 2	1454424_at	5.9
Atg7	autophagy related 7	1446633_at	5.64
Brsk2	BR serine/threonine kinase 2	1439329_a_at	5.62
Slit3	slit homolog 3 (Drosophila)	1452296_at	5.48
Mycbp2	MYC binding protein 2	1445340_at	5.48
Sod1	superoxide dismutase 1, soluble	1447761_x_at	5.26
Edn3	endothelin 3	1421136_at	5.25
Ndn	necdin	1456575_at	4.91
Tgfb2	transforming growth factor, beta 2	1423250_a_at	4.75
Slit1	slit homolog 1 (Drosophila)	1425277_at	4.65
Slitrk4	SLIT and NTRK-like family, member 4	1437744_at	4.56
Dscaml1	Down syndrome cell adhesion molecule like 1	1427392_at	4.53
Fgf8	fibroblast growth factor 8	1451882_a_at	4.3
Cdk5r2	cyclin-dependent kinase 5, regulatory subunit 2 (p39)	1450465_at	4.29
Als2	amyotrophic lateral sclerosis 2 (juvenile)	1417784_at	4.24
Mapk8ip2	mitogen-activated protein kinase 8 interacting protein 2	1418785_at	4.24
Clic5	chloride intracellular channel 5	1439505_at	4.21
Mypn	myopalladin	1435813_at	4.19
Gata3	GATA binding protein 3	1448886_at	4.08
Ephb1	Eph receptor B1	1455188_at	4.08
Nrcam	neuronal cell adhesion molecule	1458833_at	3.96
Myo3a	myosin IIIA	1431983_at	3.93
Map2	microtubule-associated protein 2	1421327_at	3.79
Epha10	Eph receptor A10	1436093_at	3.69
Brsk2	BR serine/threonine kinase 2	1431826_a_at	3.68
Chrnb2	cholinergic receptor, nicotinic, beta polypeptide 2 (neuronal)	1441837_at	3.68
Itga4	integrin alpha 4	1450155_at	3.65
Robo3	roundabout homolog 3 (Drosophila)	1436634_at	3.65
Epha4	Eph receptor A4	1439757_s_at	3.61
Elavl4	ELAV (embryonic lethal, abnormal vision, Drosophila)-like 4 (Hu antigen D)	1450258_a_at	3.61
Mapk8ip2	mitogen-activated protein kinase 8 interacting protein 2	1435045_s_at	3.57
Cdk5r1	cyclin-dependent kinase 5, regulatory subunit 1 (p35)	1433451_at	3.5

Bcl11b	B cell leukemia/lymphoma 11B	1435227_at	3.49
Epha4	Eph receptor A4	1421929_at	3.46
Pou4f2	POU domain, class 4, transcription factor 2	1437588_at	3.42
Map2	microtubule-associated protein 2	1434194_at	3.32
Myo7a	myosin VIIA	1421385_a_at	3.31
Etv4	ets variant 4	1443381_at	3.3
Sema3b	sema domain, immunoglobulin domain (Ig), short basic domain, secreted, (semaphorin) 3B	1431795_a_at	3.13
Cntnap1	contactin associated protein-like 1	1421580_at	3.13
Igf2bp1	insulin-like growth factor 2 mRNA binding protein 1	1455223_at	3.06
Grcc10	gene rich cluster, C10 gene	1429782_at	3.02
Dscam	Down syndrome cell adhesion molecule	1458625_at	-3.11
Clic5	chloride intracellular channel 5	1456873_at	-3.49
Dscaml1	Down syndrome cell adhesion molecule like 1	1432196_a_at	-3.64
Clic5	chloride intracellular channel 5	1431261_at	-3.85
Epha4	Eph receptor A4	1456863_at	-6.13
Slitrk4	SLIT and NTRK-like family, member 4	1440516_at	-6.36
Ptprz1	protein tyrosine phosphatase, receptor type Z, polypeptide 1	1418690_at	-6.47
Lhx3	LIM homeobox protein 3	1421753_a_at	-6.88
Bcl11b	B cell leukemia/lymphoma 11B	1450339_a_at	-7.77
Itga4	integrin alpha 4	1427615_at	-8.53
Zdhhc17	zinc finger, DHHC domain containing 17	1447656_at	-8.6
Edn3	endothelin 3	1441923_s_at	-10
Cacna1a	Cacna1a calcium channel, voltage-dependent, P/Q type, alpha 1A subunit	1420287_at	-11.62
